# Nodal and cripto-1: distinct functions regulate trophoblast specification in mouse pregnancy

**DOI:** 10.3389/fcell.2025.1608976

**Published:** 2025-05-19

**Authors:** Laura Girardet, Neha Kamath, Daniel Dufort

**Affiliations:** ^1^ Department of Obstetrics and Gynecology, McGill University, Montreal, QC, Canada; ^2^ Research Institute of the McGill University Health Centre, Child Health and Human Development Program, Montreal, QC, Canada

**Keywords:** Nodal signaling, cripto-1, spongiotrophoblast, giant cells, ectoplacental cone, labyrinth, placenta, pregnancy

## Abstract

**Introduction:**

Proper placentation is essential for fetal growth and development in mammals. Nodal signaling is essential to ensure proper embryo development and requires Cripto-1 as a co-receptor. Both factors have been shown to be expressed in the maternal decidua and developing placenta. Notably, a maternal loss of either Nodal or Cripto-1 leads to defective placentation resulting in intrauterine growth restriction and fetal loss. However, the role of Nodal or Cripto-1 in placental development has not been determined.

**Methods:**

To better understand the roles of Nodal and Cripto-1 in trophoblast populations, we employed a trophoblast-specific deletion model using Tat-Cre recombinant protein to induce deletion of the floxed Nodal or Cripto-1 genes exclusively in the trophectoderm at the blastocyst stage (TE-KO). Treated embryos were then transferred into the uteri of pseudopregnant mice, and implantation sites were examined at gestational days (d) 8.5 and 10.5. Placental morphology and trophoblast populations were analyzed through histological and molecular marker analysis.

**Results:**

TE-KO of Nodal led to a decrease in the implantation site size and placental thickness, primarily due to a smaller labyrinth area while the junctional zone was increased. Immunostaining revealed an important expansion of PL^+^ trophoblast giant cells and decrease of TPBPA^+^ spongiotrophoblast/glycogen cells. TE-KO of Cripto-1 also led to smaller implantation sites and reduced placental thickness, but this was attributed to a smaller junctional zone. A decrease in TPBPA^+^ spongiotrophoblast cells without affecting *Pcdh12* ^+^ glycogen cells was observed. A reduction in MCT1^+^ and *Gcm1* ^+^ syncytiotrophoblasts and an increase in total area of maternal blood sinuses within the labyrinth emphasized its disorganization. Earlier effects of Cripto-1 TE-KO on the trophoblast maintenance were witnessed at d8.5, with a marked reduction in TPBPA^+^ cells, reduced trophoblast cell proliferation (PCNA^+^) and increased apoptosis (TUNEL^+^).

**Discussion:**

The distinct phenotypes observed indicate the different roles Nodal and Cripto-1 play in placental development. This highlights the importance of other TGF-β-dependent and independent pathways involving Cripto-1. Overall, our findings highlight the critical role of Nodal and Cripto-1 in regulating key aspects of placental development, including trophoblast differentiation, cellular specification, and structural organization, promising avenues for future research in placental biology.

## 1 Introduction

Nodal signaling is a key biological pathway of the transforming growth factor (TGF-β) family and is known to be involved in early development. Since Nodal is a secreted morphogen, it acts in a concentration-dependent manner (Chen and Schier, 2001) and binds to a complex of receptors to exert its effect on target cells. Cripto-1 (also known as teratocarcinoma-derived growth factor-1, TDGF-1) is a co-receptor that forms a complex with activin type I receptors (Alk4 and Alk7) and the activin type II receptor complex (Bianco et al., 2003). Nodal binding to this complex initiates an activation cascade that leads to the phosphorylation of cytoplasmic Smad proteins, particularly Smad-2 and Smad-3. Following phosphorylation, these Smads form complexes with Smad-4 and translocate into the nucleus. Inside the nucleus, in collaboration with co-activators such as FoxH1, these complexes drive the transcriptional activation of specific target genes (Yeo and Whitman, 2001; Bianco et al., 2003). This signaling pathway is integral to developmental processes such as the initiation of gastrulation through the formation of the primitive streak (Ding et al., 1998; Yan et al., 1999; Schier, 2003). It also plays a crucial role in the patterning of the embryo, establishing the anterior/posterior and left/right axis, and orchestrating mesoderm and endoderm cell allocation. Mutations in Nodal or Cripto-1 severely affect gastrulation and lead to embryonic lethality between days 7.5–10.5 (Iannaccone et al., 1992; Zhou et al., 1993; Liguori et al., 1996; Ding et al., 1998). Both factors are expressed as early as day 3.5 in the developing blastocyst (de Castro et al., 2010; Takaoka et al., 2017).

In humans, the loss or dysregulation of uterine Nodal or Cripto-1 signaling has been associated with reproductive pathologies including endometriosis (Cruz et al., 2015), recurrent pregnancy loss (Bremm et al., 2021), pre-term delivery (Torricelli et al., 2012; Starr et al., 2018) and varying degrees of abnormal implantation (Bandeira et al., 2014; Jiang et al., 2019; Cabar et al., 2022; Ozkose et al., 2022). These findings led to the development of uterine specific Nodal and Cripto-1 conditional knockout (KO) mouse models to investigate their roles in female reproduction. The uterine specific conditional KO of both Nodal and Cripto-1 resulted in reduced fertility, as indicated by a smaller litter size and lower pregnancy rate (Park et al., 2012; Shafiei et al., 2021; Yull et al., 2023). Uterine Nodal KO females showed placental abnormalities such as abnormal labyrinth formation and an expansion of trophoblast giant cells leading to intrauterine growth restriction (Park et al., 2012). Similarly, uterine Cripto-1 KO females displayed deficiencies in placental development such as but not limited to an under-vascularized placental labyrinth, which was associated with intrauterine growth restriction and fetal mortality (Shafiei and Dufort, 2021).

Placental development in mice begins as early as day 3.5 post-fertilization with the formation of a blastocyst. The blastocyst consists of two main cell population: the inner cell mass (ICM), which gives rise to the embryo, and the trophectoderm (TE), which forms the extraembryonic tissues including the placenta (Watson and Cross, 2005). The mural TE, which is not in contact with the ICM, first differentiates into a limited number of primary trophoblast giant cells (TGC) (Rossant and Cross, 2001). These primary TGCs are invasive and vital for the remodeling of the maternal uterine stroma. Primary TGCs also contribute to the high level of proteins produced and secreted at mid gestation including placental lactogen I (PL) and proliferin (Nadra et al., 2006). Conversely, the polar trophectoderm adjacent to the ICM undergoes differentiation to form the ectoplacental cone, which expresses trophoblast specific protein alpha (TPBPA) at day 8.5 (Hu and Cross, 2011). This is followed by the development of the extraembryonic ectoderm, which will eventually form the chorion layer and fuse with the allantois to start the formation of the placental labyrinth (Cross et al., 2003). The ectoplacental cone gives rise to TGCs (Rossant and Cross, 2001) and the spongiotrophoblast layer (Jiang et al., 2023; Wu et al., 2024). As gestation progresses, glycogen trophoblast cells differentiate from the spongiotrophoblast layer and gradually infiltrate the maternal decidua in the uterine wall (Adamson et al., 2002). Subsequently, the chorion undergoes folding to form villi and create cavities where fetal blood vessels grow from the allantois (Cross et al., 2003). Around days 9.5–10.5, differentiation is initiated of chorionic trophoblast cells into two main cell types: mononuclear trophoblast cells, which surround the maternal blood sinuses, and a bilayer of multinucleated syncytiotrophoblast cells (type II). The formation of syncytiotrophoblast II cells is aided by the transcription factor glial cells missing-1 (GCM1) (Kashif et al., 2011; Jeyarajah et al., 2022). Syncytiotrophoblast II, which face the fetal vasculature, give rise to the syncytiotrophoblast I facing maternal blood (Jiang et al., 2023). In unison, the trophoblast and fetal vasculature form an extensive network within which the fetal and maternal blood flow in a concurrent manner to optimize nutrient, gas and waste exchange. By mid-pregnancy, the mouse placenta is organized into three complete layers: the maternal decidua, the junctional zone and the labyrinth. Collectively, these components work in collaboration to support fetal growth.

While the effects of Nodal and Cripto-1 constitutive KO and conditional uterine KO have been well characterized, their specific role in the trophoblast remains largely unexplored. Furthermore, both Nodal and Cripto-1 are expressed in the mouse embryonic and extraembryonic tissues. Indeed, Nodal is detected at the base of the ectoplacental cone and chorion from day 8.5 (Natale et al., 2009) and subsequently in the developing placenta, in the spongiotrophoblast layer at day 10.5 (Ma et al., 2001). Cripto-1 is expressed in embryonic and extraembryonic tissues, as well as maternal uterine cells (Liguori et al., 1996; Ding et al., 1998; Shafiei and Dufort, 2021). Given their expression in the blastocyst and extraembryonic tissues, as well as their altered expression in pregnancy complications, we hypothesize that Nodal through Cripto-1 co-receptor plays an important role in placental development by trophoblast regulation. To address this hypothesis, we have specifically knocked out Nodal or Cripto-1 in the TE, which gives rise to all trophoblast populations, and studied placental development. We demonstrate that Nodal and Cripto-1 deletion leads to two distinct placental phenotypes both of which display abnormalities.

## 2 Materials and methods

### 2.1 Animal colonies

All animal protocols and experimental procedures are compliant with the Canadian Council on Animal Care regulations and approved by the Research Institute of the McGill University Health Centre (RI-MUHC) Animal Care Committee. Wild-type CD1 mice were purchased from Charles River Laboratories. Eight-week-old wild-type CD1 males were vasectomized in accordance with RI-MUHC animal facility protocols. Vas deferens were identified, cauterized in two spots approximately 0.5 cm apart and then severed between the two cauterized spots. ROSA^mT/mG^ mice were given by H. Clarke (McGill University, Canada) and purchased from The Jackson Laboratory (Stock Number: 007,676). Mice with loxP sites flanking exon two to three of the Nodal gene (Nodal^flox/flox^) on a mixed background were generated and donated by J. Robertson (University of Oxford, UK) (Lu and Robertson, 2004). Mice with loxP sites flanking exon three to five of the Cripto-1 gene (Cripto-1^flox/flox^) on a C57BL6 background were purchased from The Jackson Laboratory (Stock Number: 016,539). The generation of this strain was previously described (Chu et al., 2005).

### 2.2 Tat-Cre deletion

Six-to eight-week-old donor Nodal^flox/flox^ or Cripto-1^flox/flox^ females were injected with 5 IU PMSG (HOR-272, Cedarlane) and 46 h later with 5 IU hCG (C1063-1 V L, Sigma) to stimulate folliculogenesis and ovulation respectively.

To optimize Tat-Cre deletion specifically in the trophectoderm, donor females were then mated with ROSA^mT/mG^ males. Mating was confirmed by the presence of a vaginal plug and designated as day 0.5 post coitum. Day 3.5 females were anesthetized and euthanized by CO_2_ asphyxiation. Mice were dissected and uteri were collected and flushed with M2 media (M7167, Millipore Sigma). Donor blastocysts were transferred into 2 consecutive droplets of KSOM (MR-106-D, Millipore Sigma) and were briefly exposed to acid Tyrode’s solution for approximately 30–45 s (T1788, Sigma). Blastocysts were then incubated in KSOM or 1.5 µM or 3 µM of Tat-Cre (SCR508, Millipore Sigma) for 2–4 h at 37 °C. Blastocysts were washed in KSOM and incubated overnight at 37°C and covered with mineral oil to prevent drying. EGFP/green (Cre recombination) and tdTomato/red (no Cre recombination) fluorescence was visualized the following morning (Inverted Fluorescent Leica DMI6000, Supplementary Figure S1).

To delete Nodal in the trophectoderm (Nodal TE-KO), Nodal^flox/flox^ donor females were mated with Nodal^flox/flox^ males, and to delete Cripto-1 in the trophectoderm (Cripto TE-KO), Cripto-1^flox/flox^ donor females were mated with Cripto-1^flox/flox^ males. The previously detailed injection protocol was followed. Exactly 24 h after donor females were first injected with PMSG, eight-to twelve-week-old CD1 recipient females were also injected with 5 IU PMSG and 46 h later with 5 IU hCG. Recipient females were then mated with vasectomized CD1 males to induce pseudopregnancy. Cripto-1^flox/flox^ blastocysts were briefly exposed to Acid Tyrode’s solution, a step that was not required for Nodal^flox/flox^ blastocysts (Supplementary Figure S1). Blastocysts were then incubated in KSOM (control, Ctl) or with 3 µM of Tat-Cre (TE-KO) for 2 h. Following incubation, blastocysts were washed in KSOM and incubated into 2 µL droplets of KSOM in preparation for transfer. Day 2.5 pseudopregnant recipient females were placed on the wire-top cage and oriented at an 80-degree angle with their rears facing upwards. A small speculum was inserted to expand the vagina and expose the cervix. A nonsurgical embryo transfer (NSET) catheter (60,010, ParaTechs Corporation) containing 10 blastocysts (either Ctl or TE-KO) was inserted into one uterine horn. Recipient females were returned to their housing cages until the gestation day of interest, either 8.5 or 10.5 days.

### 2.3 Tissue processing, sectioning, and paraffin embedding

Day 8.5 or 10.5 recipient females were anesthetized and euthanized by CO_2_ asphyxiation. Uterine horns were dissected in PBS, and whole mount images were photographed. Implantation sites were fixed in 10% neutral buffered formalin for 48 h at 4°C. Sites were dehydrated in increasing ethanol series (25%, 50%, 75%, and 100%, 20 min each) and cleared in xylene (2 × 15 min) (Leica ASP300). Sites were incubated overnight in blocks of paraffin wax (TissueTek) at 60°C and subsequently placed on cold plates to solidify for 1 h and transferred to −20°C freezer overnight for sectioning. Seven µm sections of tissue were cut with Leica RM2145 microtome before being placed on Fisherbrand Superfrost plus slides and dried at 40°C overnight.

### 2.4 Immunohistochemistry and immunofluorescence staining

Slides were deparaffinized in xylene 2 times 10 min and rehydrated in an ethanol gradient (100% twice, 95% twice, 80%, 70%, 50%, 2 min each) and twice 5 min in water. Antigen retrieval was conducted in 10 mM sodium citrate 0.05% Tween 20, pH 6, 95 °C for 20 min. Slides were cooled down for 2 min and permeabilized in TBT (TBS, 2.5% TritonX-100) for 15 min. Slides were blocked for an hour at room temperature in 10% BSA and washed 3 times 5 min with TBST (TBS, 0.025% Tween 20). Slides were incubated with a primary antibody (Supplementary Table S1) overnight at 4 °C. The following day, slides were washed twice in TBST 5 min and, for immunohistochemistry (IHC) incubated in 0.3% hydrogen peroxide solution in TBS for 20 min to block endogenous peroxidase activity. Subsequently, slides were incubated with a secondary antibody (Supplementary Table S1) for 90 min at room temperature and then washed three times 5 min with TBST. For IHC, antigen visualization slides were incubated 3,3′ diaminobenzidine for 10 min (DAB, ab642338, Abcam), then rinsed with running tap water and counterstained with hematoxylin (Gill No. 2, Sigma) for 30 s. Slides were then decolorized by briefly dipping slides in 1% acid alcohol solution. Following 5 min of rinsing in tap water, slides were briefly dipped in ammonia water bluing agent and rinsed for 5 min in tap water. Slides were then dehydrated with a reverse ethanol gradient, cleared with xylene and mounted with Permount (Fisher Scientific). For immunofluorescence (IF), slides were mounted with mowiol 4–88 (Calbiochem).

### 2.5 Hematoxylin and eosin staining

As described above for IHC, slides were deparaffinized in xylene, rehydrated in an ethanol gradient, stained for 4 min in hematoxylin and rinsed in tap water for 10 min. Slides were then decolorized by briefly dipping slides in 1% acid alcohol solution. Following 5 min of rinsing in tap water, slides were briefly dipped in ammonia water bluing agent, rinsed for 5 min in tap water and counterstained in Eosin (Sigma) for 15 s. Slides were then dehydrated, cleared with xylene and mounted with Permount.

### 2.6 *In situ* hybridization

*In situ* hybridization for *Gcm1* (probe supplied by L. Jerome-Majewska, McGill University, Canada) was conducted as previously described (Simmons et al., 2007). Slides were deparaffinized in xylene and rehydrated in an ethanol gradient. They were then treated with Proteinase K solution for 15 min, washed three times in PBS acetic anhydride 0.25% (Sigma) 5 min at room temperature. Slides were then hybridized with digoxigenin (DIG)-labeled probes (112,770 73,910, Roche) overnight at 65 °C in a humid chamber. The hybridization buffer consisted of 1× salt, 50% deionized formamide, 10% dextran sulfate, 1 mg/mL yeast tRNA (Roche), 1× Denhardt’s, diethylpyrocarbonate water and DIG-labeled probe (0.1% probe). Following overnight incubation with probes, three saline-sodium citrate washes at room temperature for 20 min were applied, followed by a RNAse A wash (0.4 M sodium chloride, 0.01 M tris, 0.005 mM EDTA, 20 μg/mL RNAse A) at 37°C for 30 min and a MABT (0.15 M sodium chloride, 0.1 M maleic acid, 0.2% tween-20) wash for 5 min at room temperature. Slides were then blocked for 1 h at room temperature in blocking solution (2% blocking reagent, goat serum, MABT) and then incubated overnight with anti-DIG antibody at 4°C. Subsequently, the slides were washed 3-times 15 min in 1× MABT and then slides were rinsed twice in NTMT (0.1 M NaCl, 0.05 M MgCl, 0.1 M tris pH 9.5, 0.1% tween-20) for 10 min and incubated in NBT/BCIP in NTMT according to the manufacturer’s instructions (Promega). Slides were counterstained with nuclear fast red, dehydrated (reverse ethanol gradient), cleared with xylene and mounted with Permount.

To assess Nodal and Protocadherin 12 (*Pcdh12)* mRNA localization, the developed RNAscope kit 2.5 H.D. (322,310, Advanced Cell Diagnostics, United States) was used in accordance with the manufacturer’s instructions with minor modifications. In brief, 7 μm formalin-fixed, paraffin-embedded tissue sections were baked, deparaffinized, treated with hydrogen peroxide followed by a 15 min target retrieval at 99 °C. Protease was incubated for 15 min, and probes for 2 h (Nodal (ACD- 436321), *Pcdh12* (ACD-489891), *DapB* negative control (ACD-310043)*, Ppib* positive control (ACD-313911)). After two washes, slides were kept at room temperature overnight. Amplification steps were pursued the next day and the fifth amplification, that can vary depending on target gene, was applied for 90 min. After detection of the signal with DAB, counterstaining and dehydration, slides were mounted with Permount.

### 2.7 TUNEL assay

Apoptosis was assessed following the manufacturer’s instructions kit (12,156,792,910, Sigma) with minor modifications. Briefly, slides were deparaffinized in xylene rehydrated in an ethanol gradient, treated with citrate sodium 0.1% TritonX-100 1% for 8 min and washed twice in PBS. Positive controls were pretreated with DNAse (2000 U/mL, Qiagen) 30 min at 37°C and negative controls were only incubated with the solution mix without enzyme. After 1 h incubation of the solution kit, slides were washed twice in PBS and mounted with Mowiol.

### 2.8 Microscopy and images analysis

Slides were imaged with an inverted epifluorescence microscope (Leica DMI6000). Fiji software (Schindelin et al., 2012) was used to generate measurements. The area of each implantation site, labyrinth, junctional zone, decidua and vasculature area was measured with the freehand tool. Placental thickness was measured from the junctional zone to the chorionic plate using three straight lines. The angle of placental implantation was determined with the angle tool from the middle of the placenta to the middle of the mesometrial uterine compartment. Areas of specific cell markers were measured after threshold adjustment. For Gcm1^+^ area, the images were also submitted to particle area analysis and only the total area was considered and reported and the labyrinth area for each site. For TUNEL and proliferating cell nuclear antigen (PCNA) analysis, all the cells within the section were counted and positive staining was assessed after threshold adjustment. Images reconstruction have been done using Adobe Photoshop CS6 13.0 with photomerge tool.

### 2.9 Statistics

Data are presented as the mean ± SEM of independent samples. For each analysis, implantation sites came from at least three different mice. Statistical analyses were conducted on GraphPad Prism 9.1.0 (California, United States). To compare experimental groups, statistical analysis was conducted using unpaired nonparametric t-tests for independent samples (with Mann-Whitney correction). Statistical significance was defined as P-values <0.05.

## 3 Results

### 3.1 Nodal controls trophoblast giant cells expansion and labyrinth development at mid-gestation

**FIGURE 1:**
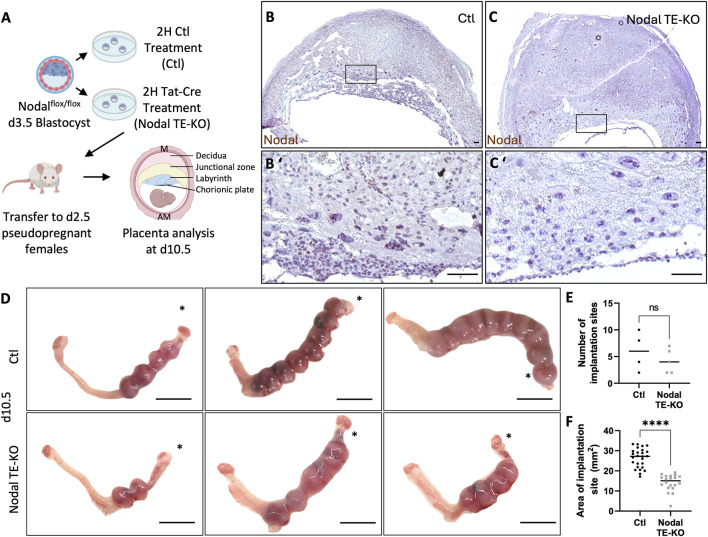
Nodal deletion in the trophectoderm results in the development of smaller implantation sites. **(A)** Control (Ctl) and Nodal trophectoderm knock-out (Nodal TE-KO) donor blastocysts were generated and transferred into pseudopregnant recipient females. Implantation sites were analyzed at day 10.5 of pregnancy (d10.5) mesometrial (M) and antimesometrial (AM) poles are showed and images are displayed following the M/AM axis. **(B, C)** The placental expression of Nodal was visualized by *in situ hybridization* in Ctl and Nodal TE-KO placentas within the labyrinth and junctional zone (n = 4 and five respectively). Scale bar = 80 µm. **(B′, C′)** Higher magnification of the areas marked by rectangles on **(B, C)**. Scale bar = 80 µm. **(D)** Uteri from Ctl and Nodal TE-KO groups. Scale bar = 1 cm. Black stars indicate the uterine horn where blastocysts were deposited by NSET. **(E)** The number of implantation sites were counted in Ctl and Nodal TE-KO groups (n = 4 and 5 respectively). **(F)** The size of implantation sites in Ctl and Nodal TE-KO was measured (n = 26 and 20 respectively). Data are presented as the mean ± SEM of independent samples. Statistical analyses were performed with t-test with Mann-Whitney correction, ns: not significant, ****p < 0.0001.

To determine the role of Nodal in trophoblast cells, we used a model where Nodal was specifically deleted in trophectoderm cells (TE) of the mouse blastocyst, which is the progenitor of all future trophoblast lineages within the placenta (Figure 1A). This was achieved using Tat-Cre, a fusion of the Cre recombinase enzyme with a cell-permeable peptide derived from HIV-TAT (TAT) and a nuclear localization sequence. This enabled the specific translocation of Cre into the nuclei of TE cells through their exposed apical membrane surface, while its inability to pass through the tight junctions restricted entry into the inner cell mass of the blastocyst, thereby achieving TE-specific deletion. Our method was first optimized on heterozygous Nodal^flox/+^ Rosa^mTmG^/^+^ embryos to visually detect Tat-Cre recombination (Supplementary Figure S1). Blastocysts with the Nodal TE deletion (TE-KO) were then transferred into the uteri of pseudopregnant CD1 female recipients. Recipient uteri of donor Nodal TE-KO blastocysts were compared to those that received control blastocysts (Ctl) exposed only to Acid Tyrode’s treatment and not Tat-Cre. Implantation sites were assessed on day 10.5, a pivotal juncture in placental development characterized by substantial trophoblast differentiation. Conditional deletion was validated by the absence or significant decrease of Nodal mRNA detected by *in situ* staining in trophoblast cells (Figures 1B, C). There was no significant difference in the number of sites between groups on day 10.5 (Figures 1D, E), but there was a 45% reduction in the implantation site size of females with Nodal TE-KO blastocysts compared to Ctl (Figure 1F). Therefore, our data shows that Nodal was effectively deleted in TE cells, and although the Nodal TE-KO did not change the rate of blastocyst implantation it reduced the overall size of implantation sites by mid-pregnancy.

**FIGURE 2:**
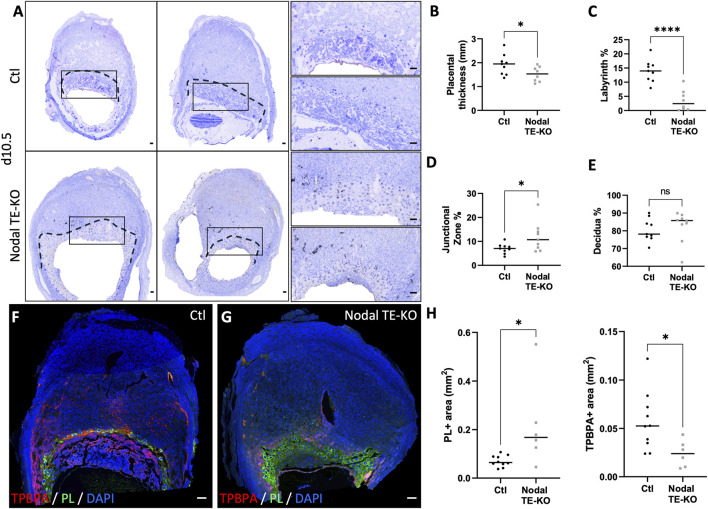
Nodal deletion in the trophectoderm results in reduced placental thickness and labyrinth layer, but an increase in the junctional zone. **(A)** Transverse sections of control (Ctl) and Nodal trophectoderm knock-out (Nodal TE-KO) implantation sites at day 10.5 of pregnancy (d10.5) displayed following mesometrial (M)/antimesometrial (AM) axis. Placenta is demarcated with a black line. Higher magnification of the areas marked by rectangles are displayed to the right. **(B)** Placental thickness (labyrinth + junctional zone) was measured in Ctl and Nodal TE-KO placenta (n = 9). **(C)** Labyrinth, **(D)** junctional zone and **(E)** decidua areas were measured as percentage of total area from mesometrial pole to chorionic plate in Ctl and Nodal TE-KO placenta (n = 9). **(F , G)** Immunofluorescent staining and **(H)** quantification of spongiotrophoblast and glycogen cells using trophoblast specific protein alpha (TPBPA) and trophoblast giant cells using placental lactogen I (PL) were performed in Ctl and Nodal TE-KO within the junctional zone and displayed following M/AM axis. (n = 10 and six respectively). Data are presented as the mean ± SEM of independent samples. Statistical analyses were performed with t-test with Mann-Whitney correction, ns: not significant, *p < 0.05, ****p < 0.0001. Scale bar = 80 µm.

As the Nodal TE-KO affected the size of implantation sites by d10.5, a more in-depth analysis of the placental layers (labyrinth, junctional zone and maternal decidua) was conducted. Our histological analyses of d10.5 placenta from Nodal TE-KO compared to the Ctl group showed a significant 20% decrease in placental thickness (Figures 2A, B). Additionally, in the Nodal TE-KO we observed a 75% decrease in the labyrinth area and 70% increase in the junctional zone. There was no difference in the maternal decidual area of the female recipients (Figures 2C–E). As the labyrinth layer was almost absent in Nodal TE-KO but an enlarged junctional zone was seen, specific cell markers of the junctional zone were used to decipher which cell types were affected in these morphologically abnormal Nodal TE-KO placentae. These markers included TPBPA, which is specific to spongiotrophoblasts, glycogen trophoblast cells and syncytiotrophoblasts, and PL, which is specific to trophoblast giant cells (TGC) (Figures 2F, G). A 3-fold increase in PL^+^ area was observed, whereas there was a 2.5-fold decrease of TPBPA^+^ area in the Nodal TE-KO (Figure 2H). Taken together, our results demonstrated that Nodal was involved in junctional zone and labyrinth development at mid-gestation. As the absence of Nodal in the TE seemed to favor PL^+^ TGC expansion and a reduction in the other TPBPA^+^ trophoblast populations, it is suggested that Nodal has a role in the regulation of trophoblast cell fate and differentiation.

### 3.2 Cripto-1 is necessary for the development of junctional zone and labyrinth trophoblast populations at midgestation

**FIGURE 3:**
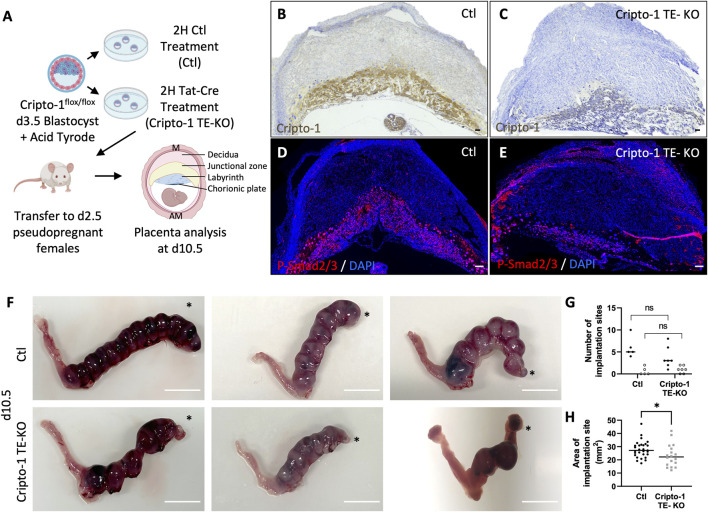
Cripto-1 deletion in the trophectoderm results in decreased Smad2/3 phosphorylation and the development of smaller implantation sites. **(A)** Control (Ctl) and Cripro-1 trophectoderm knock-out (Cripto-1 TE-KO) donor blastocysts were generated and transferred into pseudopregnant recipient females. Implantation sites were analyzed at day 8.5 or 10.5 of pregnancy (d8.5 and d10.5) mesometrial (M) and antimesometrial (AM) poles are showed and images are displayed following the M/AM axis. **(B, C)** Cripto-1 placental expression levels were visualized by immunohistochemistry in Ctl and Cripto-1 TE-KO placentas within the labyrinth and junctional zone at d10.5 (n = 7 and four respectively). Scale bar = 80 µm. **(D, E)** Immunofluorescent staining for P-Smad2/3 in Ctl and Cripto-1 TE-KO placentas within the labyrinth and junctional zone at d10.5 (n = 4 and seven respectively). Scale bar = 80 µm. **(F)** Uteri from Ctl and Cripto-1 TE-KO groups at d10.5. Scale bar = 1 cm. Black stars indicate the uterine horn where blastocysts were deposited by NSET. **(H)** The number of viable (black round) or resorbed (empty round) implantation sites were counted in Ctl and Cripto-1 TE-KO groups at d10.5 (n = 5 and seven respectively). **(G)** The size of implantation sites in Ctl and Cripto-1 TE-KO was measured at d10.5, (n = 29 and 18 respectively). Data are presented as the mean ± SEM of independent samples. Statistical analyses were performed with t-test with Mann-Whitney correction, *p < 0.05.

**FIGURE 4:**
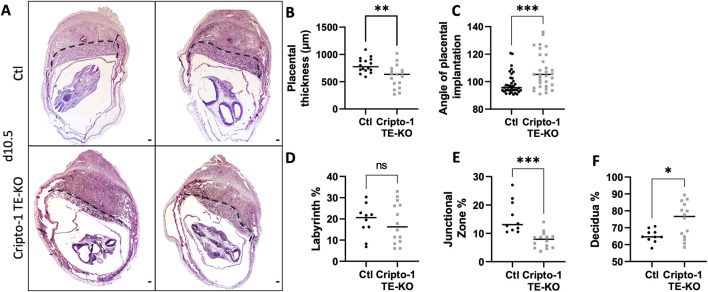
Cripto-1 deletion in the trophectoderm results in reduced placental thickness and junctional zone. **(A)** Transverse sections of control (Ctl) and Cripto-1 trophectoderm knock-out (Cripto-1 TE-KO) implantation sites at day 10.5 of pregnancy (d10.5) displayed following mesometrial (M)/antimesometrial (AM) axis. Placenta is demarcated with a black line. **(B)** Placental thickness (labyrinth + junctional zone) (n = 16 and 17) and **(C)** angle of placental implantation (n = 36 and n = 31) were measured in Ctl and Cripto-1 TE-KO placenta respectively. **(D)** Labyrinth, **(E)** junctional zone and **(F)** decidua areas were measured as percentage of total area from mesometrial pole to chorionic plate in Ctl and Cripto-1 TE-KO placenta (n = 10 and 14 respectively). Data are presented as the mean ± SEM of independent samples. Statistical analyses were performed with t-test with Mann-Whitney correction, ns: not significant, *p < 0.05; **p < 0.01; ***p < 0.001. Scale bar = 80 µm.

Cripto-1 is implicated in multiple signaling pathways during pregnancy, and is largely known to be a co-receptor for the Nodal signaling pathway (Shafiei and Dufort, 2021). To better comprehend how Nodal deletion in the TE leads to placental abnormalities, the deletion of Critpo-1 within the TE was also pursued. After optimizing the protocol on Cripto-1^flox/+^ Rosa^mTmG^/^+^ embryos (Supplementary Figure S1), Cripto-1 deletion was performed in donor Cripto-1^flox/flox^ blastocysts (Cripto-1 TE-KO), which were then transferred into pseudopregnant recipient CD1 females (Figure 3A). Day 10.5 uteri of the recipient Cripto-1 TE-KO blastocysts were compared to recipients of Ctl blastocysts. Conditional deletion of Cripto-1 was validated by the absence or significant decrease of Cripto-1 signal, as detected by IHC staining in trophoblast cells (Figures 3B, C). Additionally, loss or significant reduction of Cripto-1 was further supported by a drastic decrease in the level of p-Smad2/3, an indicator of the activated TGF-β/Nodal/Smad signaling pathway (Figures 3D,E). On day 10.5, no significant differences in the number and viability of sites between groups was observed (Figures 3F,G). Resorbed sites were observed in both control and Cripto-1 TE-KO. As this was not observed in Nodal TE-KO, it suggests that either the genetic background of the mice is more sensitive to the blastocyst treatment or that acid tyrod’s treatment had deleterious effect as observed in other studies (Fan et al., 2022). As observed in the Nodal TE-KO, Cripto-1 TE-KO led to a 20% reduction in the size of the implantation sites compared to the Ctl group (Figure 3H). Further histological analyses of d10.5 placentae from Cripto-1 TE-KO showed a significant 20% decrease in placental thickness compared to Ctl (Figures 4A, B). Placentae of Cripto-1 TE-KO appeared to be misaligned or tilted relative to the uterine antimesometrial-mesometrial axis, with an average of 10° difference in the angle of implantation compared to Ctl, with a third of them being at more than 110° (compared to 98° for Ctl) (Figure 4C). No statistically significant difference in the labyrinth area between groups was observed, with large variability in the Cripto-TE KO (Figure 4D). Additionally, in the Cripto-1 TE-KO a 65% decrease in the junctional zone and a 15% increase in the maternal decidua area was observed (Figures 4E, F). The maternal deciduae area increase could come from reduced total placental area or maternal compensation.

**FIGURE 5:**
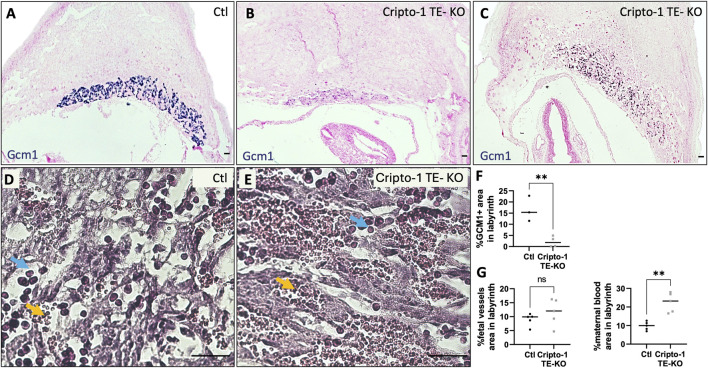
Cripto-1 deletion in the trophectoderm results in altered labyrinth organization. **(A, B, C)** *In situ* hybridization for glial cells missing-1 (*Gcm1*)*,* specific to syncytiotrophoblast-II, was performed control (Ctl) and Cripto-1 trophectoderm knock-out (Cripto-1 TE-KO) implantation sites at day 10.5 of pregnancy (d10.5) within the labyrinth and displayed following mesometrial (M)/antimesometrial (AM) axis. (n = 3 and four respectively). **(D, E)** Fetal vessels (filled with immature red blood cells, blue arrows) and maternal blood sinuses (filled with erythrocytes, yellow arrows) were visualized by hematoxylin and eosin staining in the Ctl and Cripto-1 TE-KO placental labyrinth (n = 5). **(F)** Percentage of *Gcm1* positive area, **(G)** fetal vessel area, and **(H)** area of the maternal blood sinuses relative to the total labyrinth area were quantified. Data are presented as the mean ± SEM of independent samples. Statistical analyses were performed with t-test with Mann-Whitney correction, ns: not significant, **p < 0.01. Scale bar = 80 µm.

Specific cell markers were used to identify which cell types were affected in these morphologically abnormal Cripto-1 TE-KO placentae in both the labyrinth and junctional zone. Since the labyrinth is composed mainly of syncytiotrophoblasts, we first measured *Gcm1* expression relative to the total labyrinth area due to the variability of labyrinth size between the sites (Figures 5A–C). In the Cripto-1 TE-KO, *Gcm1* positive cells revealed a disorganized labyrinth layer with less branching and a 10-fold decrease in *Gcm1* ^+^ area compared to Ctl (Figure 5F). We then examined syncytiotrophoblast I by immunofluorescence staining of monocarboxylate cotransporter 1 (MCT1), We found fewer MCT1^+^ cells in the Cripto-1 TE-KO placenta and their distribution were disbanded, in comparison to the Ctl (Supplementary Figure S2). Maternal blood sinuses and fetal vasculature network within the placental labyrinth were then examined based on the presence of nonnucleated red blood cells (maternal) and nucleated (fetal) erythrocytes respectively (Figures 5D, E **).** Although no difference in the fetal vasculature network area relative to the total labyrinth area was seen, a significant 2-fold increase was noted in the area of maternal blood sinuses relative to the total labyrinth in the Cripto-1 TE-KO group (Figure 5G), confirming an abnormal labyrinth development.

**FIGURE 6:**
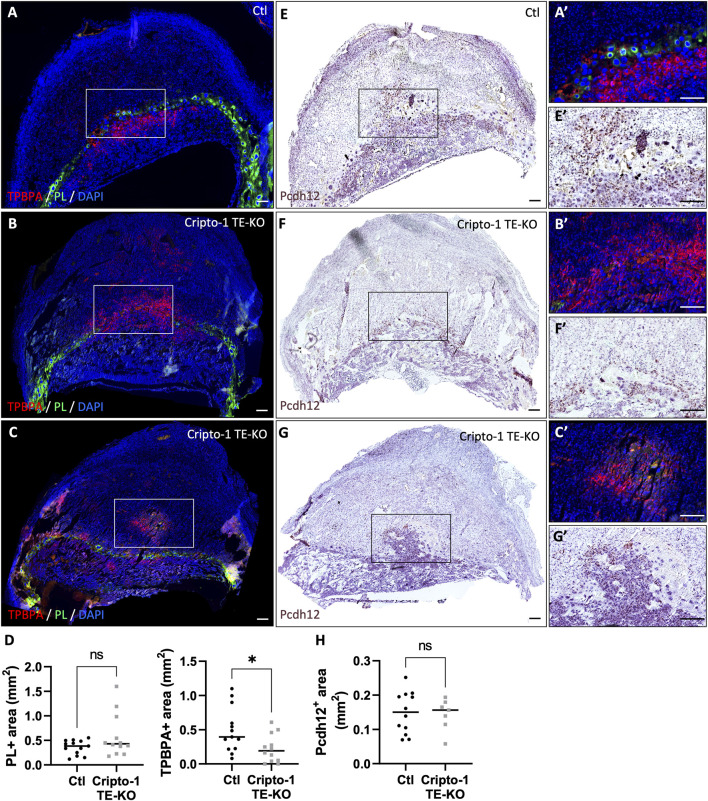
Cripto-1 deletion in the trophectoderm results in the development of an altered junctional zone with less spongiotrophoblasts. **(A, B, C)** Immunofluorescent staining and **(D)** quantification of spongiotrophoblast and glycogen cells using trophoblast specific protein alpha (TPBPA) and trophoblast giant cells using placental lactogen I (PL) were performed in control (Ctl) and Cripto-1 trophectoderm knock-out (Cripto-1 TE-KO) implantation sites at day 10.5 of pregnancy (d10.5) within the junctional zone and are displayed following mesometrial (M)/antimesometrial (AM) axis. (n = 13 and 12 respectively). **(E, F and G)** Glycogen cells were stained for Protocadherin 12 (*Pcdh12*) using RNAscope, on consecutives slides used the immunofluorescence, and **(H)** quantified in Ctl and Cripto-1 TE-KO placenta displayed following the M/AM axis (n = 12 and seven respectively). **(A′,E′,B′,F′,C′,G′**) Higher magnification of the areas marked by rectangles on **(A-G)**. Data are presented as the mean ± SEM of independent samples. Statistical analyses were performed with t-test with Mann-Whitney correction, ns: not significant, *p < 0.05. Scale bar = 80 µm.

We next examined the junctional zone, a primordial site for placental and embryo growth through the secretion of hormones and growth factors. It is composed of TGC (PL^+^), spongiotrophoblasts and glycogen trophoblasts (TPBPA^+^) (Figures 6A–C). No difference in PL^+^ TGCs was observed (Figure 6D), except for about 16% of Cripto-1 TE-KO sites where TGCs comprised most of the placenta. These mutants were designated as an extreme phenotype (Supplementary Figure S3). Interestingly, the TPBPA^+^ area was significantly reduced by 2-fold in the Cripto-1 TE-KO placentae (Figure 6D). Instead, TPBPA^+^ cells infiltrated into the maternal decidua of the Cripto-1 TE-KO rather than remaining in the junctional zone below PL^+^ cells as expected. To better differentiate between the TPBPA^+^ spongiotrophoblast or glycogen trophoblasts in this model, sequential sections were analyzed by *in situ* hybridization against *Pcdh12,* exclusive to glycogen trophoblast cells (Figures 6E–G). No differences in the *Pcdh12* ^+^ area (Figure 6H) were observed between groups. Since the decidua-localized TPBPA^+^ cells were associated with *Pcdh12* ^*+*^ signal in the Cripto-1 TE-KO, it was suggested that the decrease in TPBPA^+^ area was due to the loss of the spongiotrophoblast population, while the glycogen cell population was unaffected. Together, our data suggest that Cripto-1 is involved in junctional zone and labyrinth development likely through the regulation of syncytiotrophoblast and spongiotrophoblast populations during mid-gestation.

### 3.3 Cripto-1 controls early trophoblast development and maintenance

**FIGURE 7:**
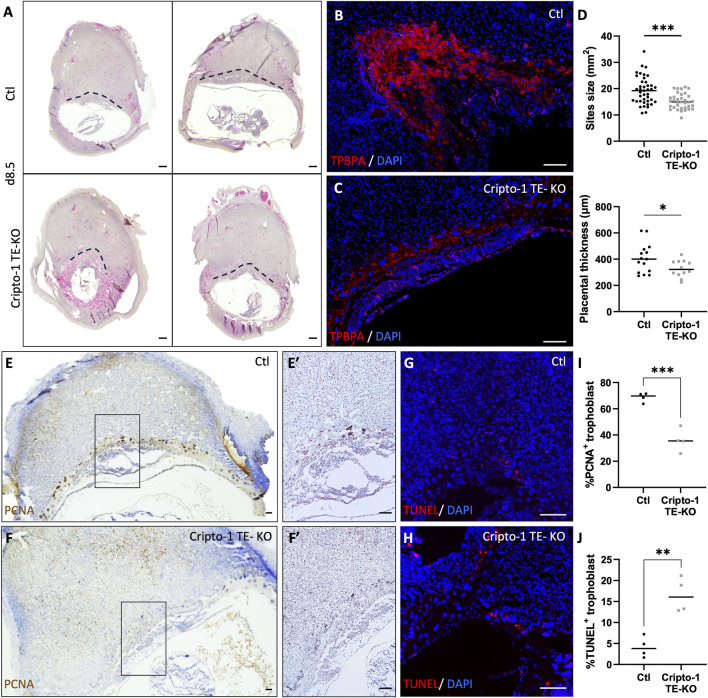
Cripto-1 deletion in the trophectoderm results in the dysregulation of early placental development and trophoblast maintenance. **(A)** Control (Ctl) and Cripto-1 trophectoderm knock-out (Cripto-1 TE-KO) donor blastocysts were generated and transferred into pseudopregnant recipient females. Implantation sites were analyzed at day 8.5 (d8.5) of pregnancy and representative transverse sections of implantations sites where placental border is demarcated with a black line are displayed following mesometrial (M)/antimesometrial (AM) axis. **(B, C)** Immunofluorescent staining of the ectoplacental cone with trophoblast specific protein alpha (TPBPA) were performed in Ctl and Cripto-1 TE-KO displayed following the M/AM axis (n = 4). **(D)** The size of the Ctl and Cripto-1 TE-KO implantation sites (n = 41 and 35 respectively), as well as placental thickness (n = 15 and 12 respectively) were measured. **(E, F)** Ctl and Cripto-1 TE-KO were stained for proliferation using proliferating cell nuclear antigen (PCNA) or **(G, H)** for cellular death with terminal deoxynucleotidyl transferase dUTP nick end labeling (TUNEL) displayed following the M/AM axis. **(E′, F′)** Enlarged imaged of PCNA staining are displayed. **(I)** Quantification of PCNA positive **(J)** quantification of TUNEL positive trophoblast are shown (n = 4). Data are presented as the mean ± SEM of independent samples. Statistical analyses were performed with t-test with Mann-Whitney correction, *p < 0.05; ***p < 0.001. Scale bar = 80 µm.

To investigate whether Cripto-1 impacts trophoblast populations through early developmental events, the placental pre-architecture at day 8.5 was studied in the Cripto-1 TE-KO. (Figure 7A; Supplementary Figure S4). To further investigate the spongiotrophoblast layer, immunofluorescence staining for TPBPA was conducted on spongiotrophoblast progenitors, which typically reside in the ectoplacental cone at day 8.5 (Figures 7B, C). A drastic reduction of the TPBPA^+^ population could be observed, indicating disrupted early cellular organization in the Cripto-1 TE-KO. Similar to day 10.5, no differences in the overall number and viability of implantation sites were seen (Supplementary Figure S4). However, a 30% decrease in the size of the implantation sites and placental thickness was observed in Cripto-1 TE-KO compared to Ctl on day 8.5 (Figure 7D). To determine the reason for the decrease in the TPBPA^+^ population, we assessed proliferation by immunostaining against proliferating cell nuclear antigen (PCNA) (Figures 7E, F) and apoptosis was examined by terminal deoxynucleotidyl transferase dUTP nick end labeling assay (TUNEL) (Figures 7G, H). Proliferation decreased by 2-fold in the Cripto-1 TE-KO placentae compared to Ctl (Figure 7I). We separated giant cells from other trophoblast cells for proliferation analysis, and both displayed the same decrease in proliferation. This result could suggest a common regulation of all trophoblasts by Cripto-1 (Supplementary Figure S4). Furthermore, apoptosis increased by 4-fold in Cripto-1 TE-KO placentae compared to Ctl (Figure 7J). Overall, these results suggested an early role for Cripto-1 in trophoblast maintenance and differentiation during mouse pregnancy.

## 4 Discussion

Placentation is critical for sustaining pregnancy, yet the mechanisms of Nodal signaling and Cripto-1 in placental development remains unclear. The deletion of Nodal and Cripto-1 in the TE was necessary to investigate their specific roles in trophoblast populations during placental development. To date, the lack of a Cre-expressing mouse line designed for TE-specific gene deletion hindered the direct investigation into placental effects. Only one study so far has used shRNA in blastocysts to decrease Cripto-1 expression by 50%, but this approach was limited to day 4.5 of pregnancy (Gershon et al., 2018). Here, the Tat-Cre recombinant protein was chosen for its ability to utilize the natural permeability of the TE apical membrane. This strategy enables specific recombination of loxP-flanked genomic sequences in trophoblast-specific contexts, while avoiding potential safety concerns associated with lentiviral vector-mediated gene delivery (Georgiades et al., 2007; Ozguldez et al., 2020). It was noted that although recombination efficiency was high (94% and 93% for deletion of Nodal and Cripto-1 in the TE respectively), some blastocysts still contained non-recombined cells in the TE possibly due to differences in cell permeability, suggesting a possible heterogeneity and phenotypic variability in our TE-KO models. Overall, this approach allowed for efficient and specific gene knockout in the TE, providing a valuable tool for studying the role of Nodal and Cripto-1 in placental development and overcoming limitations of previous methods.

**FIGURE 8:**
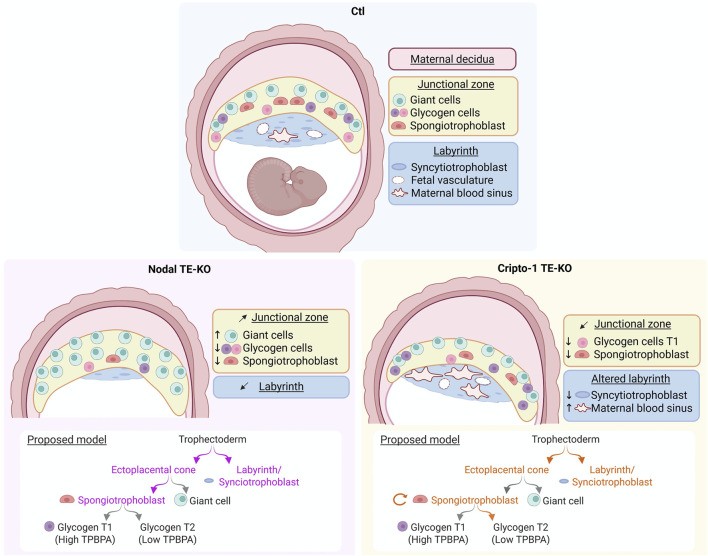
Nodal and Cripto-1 are key mediators of placental development and have distinct roles in trophoblast specification during mouse pregnancy. (Upper panel) Following mesometrial (M)/antimesometrial (AM) axis a normal implantation site (Ctl) at mid-pregnancy is contrasted to the specific findings following (Lower left panel) Nodal deletion and (Lower right panel) Cripto-1 deletion in the trophectoderm (TE-KO). A model for Nodal (violet) and Cripto-1 (orange) roles in trophoblast specification is proposed below each panel. Image created on BioRender. (Girardet, 2025; ug7bbf3).

Our study identifies both factors as key players of placental development through the regulation of various trophoblast populations (Figure 8). Nodal deletion in the TE leads to a smaller implantation site at mid-gestation, with the placental junctional zone containing mostly PL^+^ giant cells. This finding could indicate a critical role for Nodal in spongiotrophoblast differentiation. Furthermore, the important decrease in labyrinth size could also indicate an early role for Nodal in trophectoderm differentiation. Interestingly, the Cripto-1 TE-KO did not recapitulate the Nodal TE-KO phenotype. Although implantation sites were also smaller than Ctl, the Cripto-1 TE-KO placenta appeared tilted, and a thinner junctional zone was caused by a decrease in spongiotrophoblast population. There was also altered labyrinth branching due to a decrease in syncytiotrophoblasts and an increase in maternal blood spaces. Furthermore, at 8.5 days of pregnancy, Cripto-1 deletion also led to a smaller ectoplacental cone (TPBPA^+^) accompanied by a decrease in proliferation and an increase in apoptosis. Thus, Cripto-1 seems to be essential for early trophoblast maintenance within the ectoplacental cone and spongiotrophoblast maintenance at midgestation.

Nodal signaling is mostly known to be a necessary for axis specification in early embryo development. Nodal embryonic hypomorphic mutants have disrupted gastrulation but also aberrant placental development (Iannaccone et al., 1992; Zhou et al., 1993), displaying an increase in polyploid giant cells and a loss of the spongiotrophoblast cell and labyrinth layer, which is very similar to our Nodal TE-KO. Furthermore, uterine Nodal KO females also showed abnormal labyrinth formation and an expansion of trophoblast giant cells in line with our results (Park et al., 2012). When Rcho (trophoblast stem cell line derived from rat choriocarcinoma) cells and mouse trophoblasts are transfected with the Nodal gene, the PL promoter was inhibited leading a decrease in giant cell differentiation (Ma et al., 2001). Therefore, both the promotion of spongiotrophoblast differentiation and inhibition of giant cell differentiation could be regulated by Nodal. Similarly, a hypomorphic Nodal mutant displayed an increase in giant cells, reduced labyrinth and an expansion of spongiotrophoblast (Ma et al., 2001). However, the expansion of spongiotrophoblast described by the authors could also be only an expansion of glycogen cells, as the 4,311 marker used for spongiotrophoblast has now been shown to also be expressed by glycogen cells (Coan et al., 2006).

To date, the Cripto-1 co-receptor has been shown to be necessary for Nodal signaling (Yeo and Whitman, 2001; Yan et al., 2002; Watanabe et al., 2007). However, the Cripto-1 TE-KO did not entirely recapitulate Nodal TE-KO phenotype. Therefore, either Nodal could act independently of Cripto-1 or other functions of Cripto-1 could compensate for the loss of Nodal signaling. Indeed, Cripto-1 functions through both TGF-β dependent and independent pathways as either as a co-receptor in a membrane-bound form (cis) or as a ligand in a soluble form (trans) (Yan et al., 2002). As a co-receptor for Nodal, it is mainly known to strongly enhance Nodal signaling when membrane-bound (Reissmann et al., 2001; Ben-Haim et al., 2006; Liguori et al., 2008), and to a lesser extent in soluble form (Yan et al., 2002; Watanabe et al., 2007). Cripto-1 TGF-β independent signaling pathways could also be at play in the Cripto-1 TE-KO model. Three other roles of Cripto-1 have been described so far. Firstly, Cripto-1 can interact with GPC1 (Bianco et al., 2003) and GRP78 (Gray et al., 2003) further activating SRC/mitogen-activated protein kinase (MAPK)/phosphatidylinositol 3-kinase (PI3K)/AKT pathways and regulating cell growth and survival. In the study by Gershon et al., shRNA in blastocyst decreased Cripto-1 expression by 50% and led to both Smad2/3 and SRC/AKT phosphorylation diminution suggesting that both TGF-β and c-Src/MAPK/AKT pathways are important at this early stage of pregnancy (Gershon et al., 2018). At later stages of gestation in the mouse placenta, spatial clustering indicated that *Gpc1* and *Grp78* were expressed in 14% and 90% of the placental cells at day 8.5 respectively, and became more specific to the spongiotrophoblast and glycogen cells at day 10.5 (Wu et al., 2024) underlying their possible roles at mid-gestation. Secondly, Cripto-1 can also stabilize components of the Wnt signaling pathway (Nagaoka et al., 2013; Lo et al., 2018), which during placental development regulates cell proliferation and differentiation, including the critical differentiation and maintenance of the TGC layer and syncytiotrophoblasts (Dietrich et al., 2022). Thirdly, Cripto-1 can regulate the Notch signaling pathway through facilitating the posttranslational maturation of Notch receptors (Watanabe et al., 2009). In the placenta, Notch signaling has been shown to regulate trophoblast fate and maintenance (Luo et al., 2020; Perlman et al., 2021) but is not required for mouse placental formation (Shawber et al., 2019). Thus, in the Cripto-1 TE-KO model, c-Src/MAPK/AKT pathways, through GPC1 and GRP78, Wnt and Notch signaling could also explain the variable phenotype. Network analysis and gene ontology indicates that the Cripto-1 gene family is closely linked to key pregnancy pathways, including TGF-β, c-Src/MAPK/AKT, Notch, but also TNFα, IFNγ, and IL-6 (Bremm et al., 2021), so other unknown factors regulated by Cripto-1 could also be implicated.

Furthermore, in alignment with our results, the uterine conditional KO of Cripto-1 resulted in defective placentation, marked by reduced vascularization in the placental labyrinth, which ultimately led to intrauterine growth restriction and fetal mortality (Shafiei and Dufort, 2021). Interestingly, maternal uterine conditional KO of Cripto-1 led to reduced fetal vasculature, but here the Cripto-1 TE-KO led to an increase in maternal vasculature, suggesting a role for Cripto-1 in the balance of labyrinth formation. This could be explained by cellular crosstalk within the placenta and/or compensation by fetal or maternal soluble secreted form of Cripto-1, as Cripto-1 is not only expressed in TE-derived cells but also in maternal uterine stromal cells and decidual cells (Shafiei et al., 2021). Thus, in our model, the maternal decidua adjacent to the developing placenta could upregulate the production and secretion of cleaved Cripto-1 to compensate for its absence in TE-derived trophoblast populations.

The junctional zone in Cripto-1 TE-KO placentas showed the most pronounced defects in terms of compartment size and the comprising cell types. In 85% of the viable implantation sites at day 10.5, less TPBPA staining but no difference in *Pcdh12* marker was observed suggesting a specific decrease in the spongiotrophoblast layer but not glycogen cells. This finding was not reported with the uterine knock-out mice model (Shafiei et al., 2021), suggesting a specific role the Cripto-1 membrane-bound form within those cells. *Pcdh12* ^+^ glycogen trophoblast cells also showed an increase in TPBPA staining, which could indicate a less differentiated state. Conversely, other studies have identified two distinct populations of glycogen cells on day 10.5 by single cell RNA sequencing, both equivalent in proportion but each expressing either *Pcdh12* or *Tpbpa* at a higher level (Wu et al., 2024). Therefore, this could also represent a change in glycogen cell type. In 16% of Cripto-1 TE-KO implantation sites an increase in PL^+^ TGCs was observed, which aligns with poor maintenance of the spongiotrophoblast layer, favoring differentiation into TGCs or glycogen cells and resembles the Nodal TE-KO phenotype. This difference between placental phenotypes could stem from the efficiency of Cripto-1 deletion in our model, a difference in maternal Cripto-1 compensation or the implication of other signaling pathways as discussed above. Together, these results suggest that Cripto-1 regulates the fate of ectoplacental derived trophoblasts, favoring spongiotrophoblast maintenance. This was further supported by proliferation and apoptosis assays revealing a reduction in TPBPA^+^ trophoblast cells earlier on day 8.5 of pregnancy. Our findings suggest an overlap in the role of Nodal and Cripto-1 in placental development, while also influencing other signaling pathways requiring Cripto-1.

In conclusion, this study offers comprehensive insights into the role of Nodal and Cripto-1 in placental development using TE-KO models. Our results indicate that Cripto-1 likely plays a continuous role in regulating processes critical for placental growth and maintenance throughout early development and implicates a multitude of signaling pathways. Importantly, these findings not only deepen our understanding of fundamental placental processes but also have significant implications for reproductive health, particularly regarding pregnancy complications in humans. Ultimately, identifying Nodal and Cripto-1 as critical regulators in placentation opens promising avenues for future research and clinical applications in reproductive medicine.

## Data Availability

The original contributions presented in the study are included in the article/Supplementary Material, further inquiries can be directed to the corresponding authors.
