# Supplementary material for: Nodal and cripto-1: distinct functions regulate trophoblast specification in mouse pregnancy
Source: Front Cell Dev Biol. 2025 May 19;13:1608976. doi: 10.3389/fcell.2025.1608976 (PMC12127310; doi:10.3389/fcell.2025.1608976)
Supplement: Supplementary file 1 [file DataSheet1.docx]

Supplementary Material

Nodal and Cripto-1: distinct functions regulate trophoblast specification in mouse pregnancy

Girardet Laura^1,2^, Kamath Neha^1,2^, Dufort Daniel^1,2,*^

^1^Department of Obstetrics and Gynecology, McGill University, Montreal, QC, H4A 3J1, Canada.

^2^Research Institute of the McGill University Health Centre, Child Health and Human Development Program, Montreal, QC, H4A 3J1, Canada.

***Correspondence:**Daniel Dufort
daniel.dufort@mcgill.ca

Keywords: Nodal signaling, Cripto-1, Spongiotrophoblast, Giant cells, Ectoplacental cone, Labyrinth, Placenta, Pregnancy.

# Supplementary Table

| **Name** | **Type** | **Host species** | **Dilution, IF or IHC** | **Company** | **Catalog number** |
| --- | --- | --- | --- | --- | --- |
| Cripto-1 | Primary, polyclonal | Rabbit | 1-200, IHC | Abcam | ab19917 |
| DIG | Primary, polyclonal | Sheep | 1-1000, *in situ* | Roche | 11093274910 |
| Donkey anti-goat 488 | Secondary | Donkey | 1-300, IF | Invitrogen | A11055 |
| Goat anti-mouse HRP | Secondary | Goat | 1-200, IHC | Promega | W4021 |
| Goat anti-chicken 633 | Secondary | Goat | 1:300, IF | ThermoFisher | A-21103 |
| Goat anti-rabbit 594 | Secondary | Goat | 1-75, IF | Invitrogen | R37119 |
| Goat anti-rabbit HRP | Secondary | Goat | 1-200, IHC | Invitrogen | 31460 |
| MCT1 | Primary, polyclonal | Chicken | 1-100, IF | Sigma | AB1286-I |
| P-Smad2/3 | Primary, polyclonal | Rabbit | 1:100, IF | Santacruz | Sc11769 |
| PCNA | Primary, monoclonal | Mouse | 1-50, IF | Santacruz | sc56 |
| PL | Primary, polyclonal | Goat | 1-200, IF | Santa Cruz | sc34713 |
| TPBPA | Primary, polyclonal | Rabbit | 1-200, IF | Abcam | ab104401 |

**Supplementary Table 1**. Information regarding primary and secondary antibodies used in this study.

# Supplementary Figures


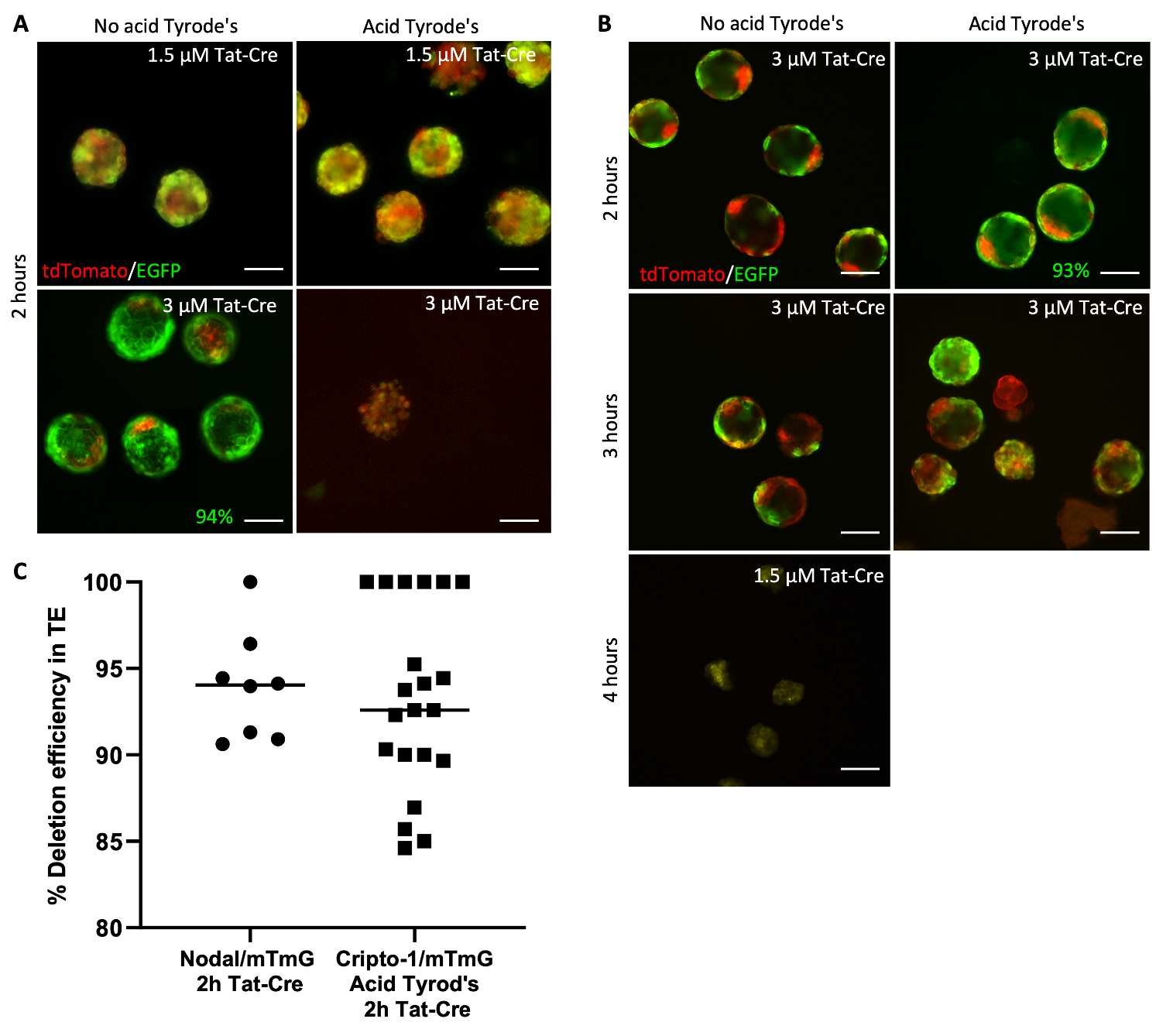


**Supplementary Figure 1.** **Optimization of Tat-Cre treatment for TE-specific Nodal or Cripto-1 deletion. (A)** Nodal deletion in the trophectoderm (Nodal TE-KO) was validated by donor blastocysts heterozygous for the mTmG and Nodal loxP alleles. Importantly, the mTmG reporter indicates tdTomato before recombination and EGFP after Cre recombination. Blastocysts were treated with or without acid Tyrode to further permeabilize the zona pellucida, followed by a 2-hour incubation with 1.5 to 3 μM of Tat-Cre (n=3 independent experiments). Optimal Nodal TE-KO was achieved without acid Tyrode’s treatment, followed by a 2h incubation with 3 μM of Tat-Cre, and confirmed by positive EGFP staining. The ICM did not have recombination as shown by tdTomato. **(B)** Cripto-1 deletion in trophectoderm (Cripto-1 TE-KO) was performed on donor blastocysts heterozygous for the mTmG and Cripto-1 loxP alleles. Blastocysts were treated with or without acid Tyrode, followed by a 2-, 3- or 4-hour incubation with 1.5 to 3 μM of Tat-Cre (n=3 independent experiments). Effective Cripto-1 TE-KO was achieved using acid Tyrode’s treatment, followed by a 2h incubation with 3 μM of Tat-Cre and confirmed by positive EGFP staining. The ICM did not have recombination as shown by tdTomato. **(C)** Effective recombination for each model was counted based on positive EGFP TE reported to the total TE (EGFP or TdTomato stained). Scale bar = 80 µm.


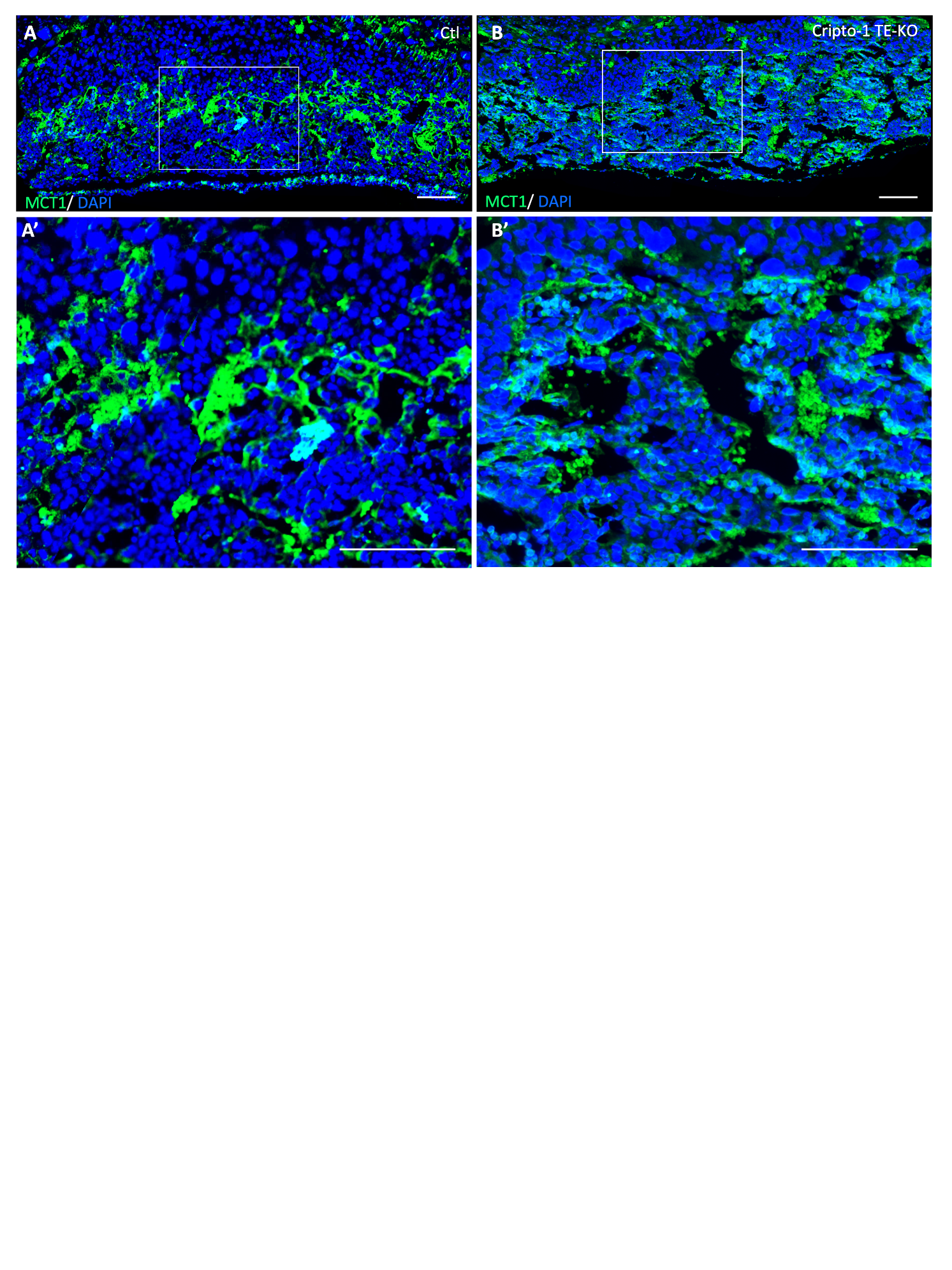


**Supplementary Figure 2.** **Cripto-1 deletion in the trophectoderm results in an altered labyrinth displaying less syncytiotrophoblast-I. (A)** Control (Ctl) and **(B)** Cripto-1 trophectoderm knock-out (Cripto-1 TE-KO) implantation sites were stained for monocarboxylate cotransporter 1 (MCT1) positive cells, specific to syncytiotrophoblast-I at day 10.5 of pregnancy within the labyrinth. Images are displayed following the mesometrial (M) / antimesometrial (AM) axis. (n=4 and 7 respectively). **(A’,B’)** Higher magnification of the areas marked by rectangles on **A,B.** Scale bar = 80 µm.


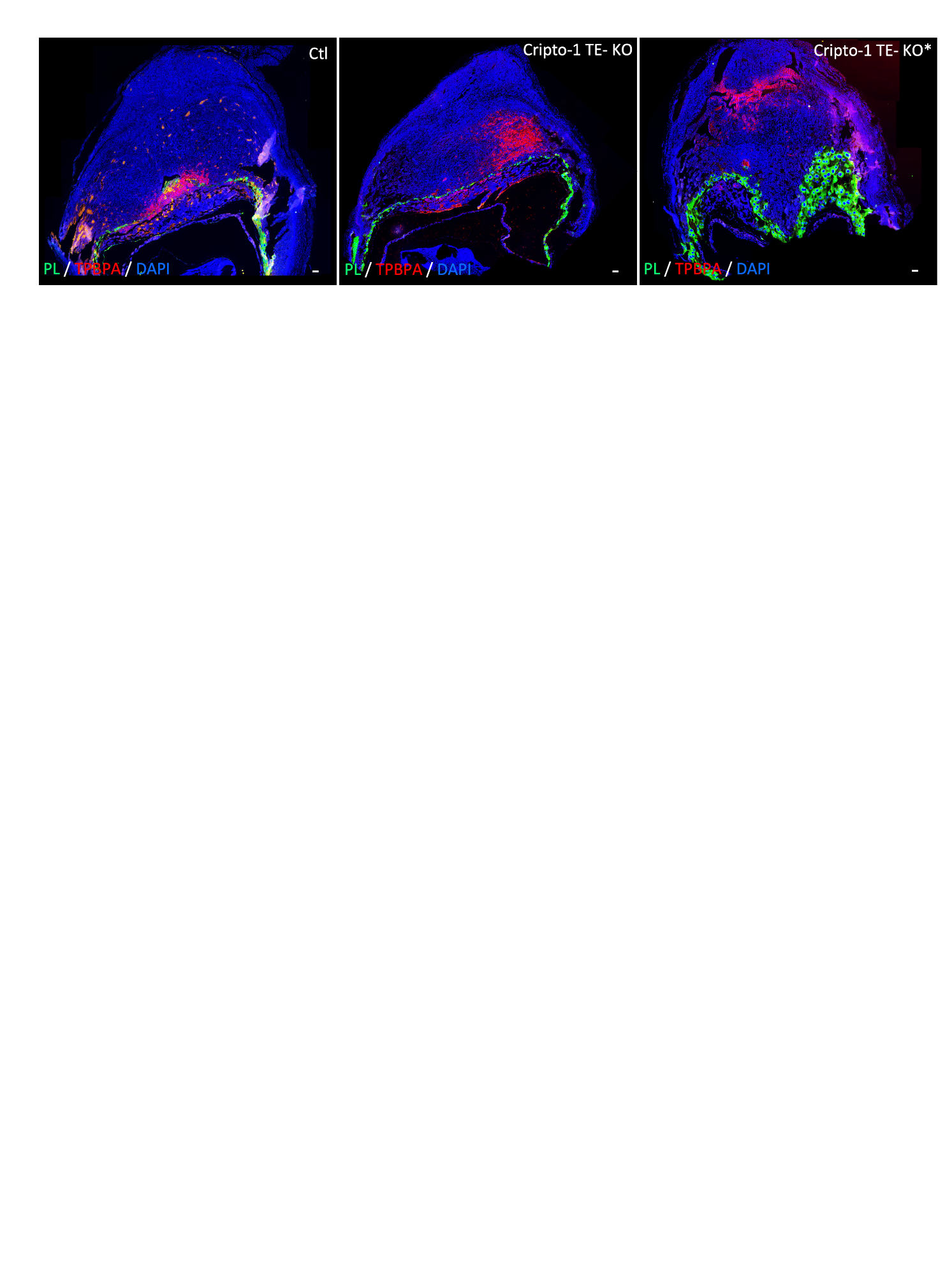


**Supplementary Figure 3.** **Cripto-1 deletion in the trophectoderm results in a range of junctional zone phenotypes.** Immunofluorescent staining of spongiotrophoblast and glycogen cells with trophoblast specific protein alpha (TPBPA) and trophoblast giant cells with placental lactogen I (PL) were performed in control (Ctl) and Cripto-1 trophectoderm knock-out (Cripto-1 TE-KO) on implantation sites at day 10.5 of pregnancy within the junctional zone. Images are displayed following the mesometrial (M) / antimesometrial (AM) axis. (n=13 and 12 respectively). An extreme phenotype was observed in 4 out of 24 sites (approximately 16%) and distinguished as Cripto-1 TE-KO*. Scale bar = 80 µm.


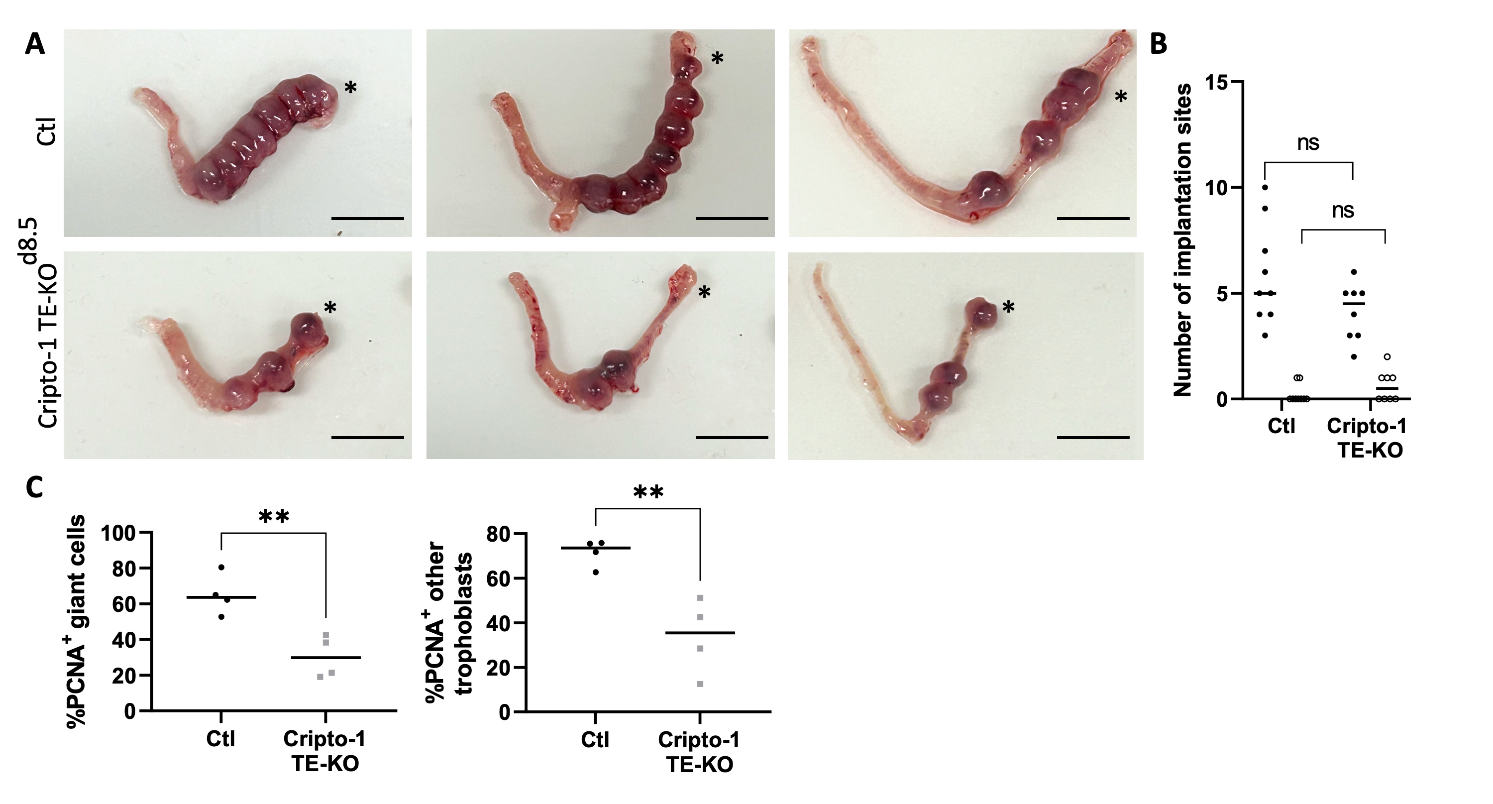


**Supplementary Figure 4.** **Cripto-1 deletion in the trophectoderm results in smaller implantation sites with dysregulated trophoblast maintenance at day 8.5. (A)** Control (Ctl) and Cripto-1 trophectoderm knock-out (Cripto-1 TE-KO) donor blastocysts were generated and transferred into pseudopregnant recipient females. Implantation sites were analyzed at day 8.5 of pregnancy. Representative images of uteri are displayed; black stars indicate the horn in which blastocysts were deposited using NSET. **(B)** The number of viable (black round) or resorbed (empty round) implantation sites were counted for Ctl and Cripto-1 TE-KO (n=9 and 8 respectively). **(C)** Ctl and Cripto-1 TE-KO were stained for proliferation using proliferating cell nuclear antigen (PCNA) and quantification of PCNA positive giant cell or other trophoblasts were distinguished (n=4). Data are presented as the mean ± SEM of independent samples. Statistical analyses were performed with t-test with Mann-Whitney correction **p < 0.01. Scale bar = 1 cm.
